# Supplementary material for: The impact of removing financial incentives and/or audit and feedback on chlamydia testing in general practice: A cluster randomised controlled trial (ACCEPt-able)
Source: PLoS Med. 2022 Jan 4;19(1):e1003858. doi: 10.1371/journal.pmed.1003858 (PMC8726492; doi:10.1371/journal.pmed.1003858)
Supplement: S1 Fig — (PDF) [file pmed.1003858.s002.pdf]

S1 Figure: Annual chlamydia testing rates for ACCEPt into *ACCEPt-able*[a]

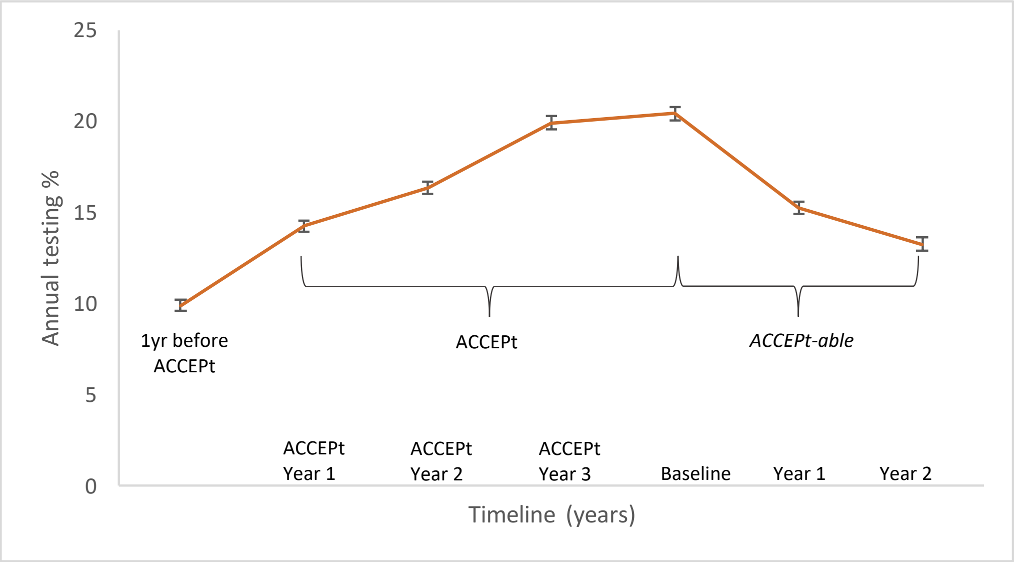

[a]Error bars correspond to 95% confidence intervals
