# Supplementary material for: The impact of removing financial incentives and/or audit and feedback on chlamydia testing in general practice: A cluster randomised controlled trial (ACCEPt-able)
Source: PLoS Med. 2022 Jan 4;19(1):e1003858. doi: 10.1371/journal.pmed.1003858 (PMC8726492; doi:10.1371/journal.pmed.1003858)
Supplement: S1 Table — (DOCX) [file pmed.1003858.s003.docx]

### S1 Table: Primary outcome chlamydia testing by sex and age group, factorial analysis

|  |  | Removal of incentive payments (intervention) (Groups A+C) | | | Retain incentive payments (control)  (Groups B+D_ | | Treatment effect  within subgroup level^c^ | | Interaction test^d^ |
| --- | --- | --- | --- | --- | --- | --- | --- | --- | --- |
|  |  | n/N | Testing rate  % (95%CI) | | n/N | Testing rate  % (95%CI) | Effect (95% CI) | p value | p value |
| Male | Baseline^a^ | 1204/9623 | 12.5 (11.1 to 13.9) | | 1565/10966 | 14.3 (12.1 to 16.4) | Diff: -0.3 (-2.9 to 2.2)  OR: 0.8 (0.6 to 1.2) | 0.7922  0.2689 | Difference: 0.0664  OR: 0.9946 |
|  | Year 1 | 686/9054 | 7.6 ( 6.7 to 8.5) | | 1185/11005 | 10.8 ( 7.8 to 13.7) |  |  |  |
|  | Year 2 | 408/6145 ^b^ | 6.6 ( 5.4 to 7.9) | | 780/ 8441^b^ | 9.2 ( 6.3 to 12.1) |  |  |  |
| Female | Baseline^a^ | 3388/13139 | 25.8 (23.3 to 28.3) | | 3952/15797 | 25.0 (22.1 to 27.9) | Diff: -2.8 (-6.5 to 1.0)  OR: 0.8 (0.6 to 1.1) | 0.1473  0.1749 |  |
|  | Year 1 | 2346/12230 | 19.2 (16.9 to 21.4) | | 3107/15747 | 19.7 (16.0 to 23.4) |  |  |  |
|  | Year 2 | 1312/8506 ^b^ | 15.4 (12.7 to 18.1) | | 2229/12635 b | 17.6 (13.0 to 22.3) |  |  |  |
| Age 16-19 years | Baseline^a^ | 1357/6985 | 19.4 (16.8 to 22.0) | | 1648/8220 | 20.0 (15.1 to 25.0) | Diff: -1.9 (-5.1 to 1.3)  OR: 0.8 (0.6 to 1.1) | 0.2382  0.1896 | Difference: 0.7907  OR: 0.9520 |
|  | Year 1 | 864/6526 | 13.2 (11.4 to 15.1) | | 1314/8233 | 16.0 (10.9 to 21.0) |  |  |  |
|  | Year 2 | 489/4463 ^b^ | 11.0 ( 8.8 to 13.1) | | 855/6216^b^ | 13.7 ( 7.6 to 19.9) |  |  |  |
| Age 20-24 years | Baseline^a^ | 1891/8047 | 23.5 (21.4 to 25.6) | | 2234/9517 | 23.5 (20.9 to 26.1) | Diff: -2.1 (-6.0 to 1.7)  OR: 0.8 (0.6 to 1.1) | 0.2789  0.2187 |  |
|  | Year 1 | 1271/7522 | 16.9 (14.5 to 19.3) | | 1775/9505 | 18.7 (15.2 to 22.1) |  |  |  |
|  | Year 2 | 719/5182 ^b^ | 13.9 (11.5 to 16.2) | | 1238/7561^b^ | 16.4 (12.7 to 20.1) |  |  |  |
| Age 25-29 years | Baseline^a^ | 1344/7730 | 17.4 (15.7 to 19.1) | | 1635/9026 | 18.1 (16.3 to 20.0) | Diff: -1.2 (-4.3 to 1.9)  OR: 0.8 (0.6 to 1.2) | 0.4527  0.2880 |  |
|  | Year 1 | 897/7236 | 12.4 (11.0 to 13.8) | | 1203/9014 | 13.3 (11.2 to 15.5) |  |  |  |
|  | Year 2 | 512/5006 ^b^ | 10.2 ( 8.5 to 12.0) | | 916/7299^b^ | 12.5 ( 9.2 to 15.9) |  |  |  |
|  |  | Removal of audit+feedback (intervention)  (Groups B+D) | | | Retain audit+feedback (control)  (Groups A+D) | | Treatment effect  within subgroup level^c^ | | Interaction test^d^ |
|  |  | n/N | | Testing rate  % (95%CI) | n/N | Testing rate  % (95%CI) | Effect (95% CI) | p value | p value |
| Male | Baseline^a^ | 1396/10093 | | 13.8 (11.7 to 16.0) | 1373/10496 | 13.1 (11.3 to 14.9) | Diff: -3.0 (-5.6 to -0.4)  OR: 0.6 (0.4 to 0.8) | 0.0233  0.0017 | Difference: 0.6087  OR: 0.0078 |
|  | Year 1 | 816/ 9743 | | 8.4 ( 6.5 to 10.3) | 1055/10316 | 10.2 ( 7.4 to 13.1) |  |  |  |
|  | Year 2 | 402/ 6510 ^b^ | | 6.2 ( 4.7 to 7.6) | 786/ 8076 b | 9.7 ( 7.1 to 12.4) |  |  |  |
| Female | Baseline^a^ | 3539/13417 | | 26.4 (23.8 to 29.0) | 3801/15519 | 24.5 (21.7 to 27.3) | Diff: -3.7 (-7.6 to -0.2)  OR: 0.8 (0.6 to 1.0) | 0.0651  0.0981 |  |
|  | Year 1 | 2513/12995 | | 19.3 (17.2 to 21.5) | 2940/14982 | 19.6 (15.7 to 23.5) |  |  |  |
|  | Year 2 | 1407/9133 ^b^ | | 15.4 (12.5 to 18.3) | 2134/12008^b^ | 17.8 (13.1 to 22.5) |  |  |  |
| Age 16-19 years | Baseline^a^ | 1429/7202 | | 19.8 (17.5 to 22.2) | 1576/8003 | 19.7 (14.5 to 24.9) | Diff: -4.2 (-7.4 to -1.1)  OR: 0.6 (0.5 to 0.9) | 0.0085  0.0065 | Difference: 0.1702  OR: 0.1842 |
|  | Year 1 | 949/6917 | | 13.7 (11.9 to 15.6) | 1229/7842 | 15.6 (10.4 to 21.0) |  |  |  |
|  | Year 2 | 466/4669 ^b^ | | 10.0 ( 7.9 to 12.1) | 878/6010^b^ | 14.6 ( 8.5 to 20.7) |  |  |  |
| Age 20-24 years | Baseline^a^ | 2007/8285 | | 24.2 (21.3 to 27.1) | 2118/9279 | 22.8 (20.9 to 24.7) | Diff: -4.0 (-7.8 to -0.2)  OR: 0.7 (0.5 to 1.0) | 0.0417  0.0252 |  |
|  | Year 1 | 1375/8014 | | 17.2 (14.8 to 19.6) | 1671/9013 | 18.5 (15.0 to 22.1) |  |  |  |
|  | Year 2 | 736/5507 ^b^ | | 13.4 (10.8 to 15.9) | 1221/7236^b^ | 16.9 (13.3 to 20.4) |  |  |  |
| Age 25-29 years | Baseline^a^ | 1499/8023 | | 18.7 (16.5 to 20.8) | 1480/8733 | 16.9 (15.6 to 18.3) | Diff: -1.6 (-5.0 to 1.8)  OR: 0.8 (0.6 to 1.2) | 0.3488  0.3368 |  |
|  | Year 1 | 1005/7807 | | 12.9 (10.7 to 15.0) | 1095/8443 | 12.9 (11.2 to 14.7) |  |  |  |
|  | Year 2 | 607/5467 ^b^ | | 11.1 ( 8.4 to 13.8) | 821/6838^b^ | 12.0 ( 8.8 to 15.2) |  |  |  |

Diff = Absolute difference. OR = Odds Ratio. n=number tested aged 16 to 29 years; N=number of individuals aged 16 to 29 years attending the clinic. ^a^Baseline = the 12-month period prior to randomisation. Year 1 = 1-12 months after randomisation. Year 2 = 13-24 months after randomisation; ^b^ Numerator and denominator less than for Baseline and Year 1 because not all clinics contributed 12 months of data to year 2. ^c^Models account for minimisation variables including annual chlamydia testing rates among 16-29 year olds and number of 16-29 year olds attending the clinic each year. ^d^Interaction test of whether treatment effect is different between males and females or between age groups.
