# Supplementary material for: The impact of removing financial incentives and/or audit and feedback on chlamydia testing in general practice: A cluster randomised controlled trial (ACCEPt-able)
Source: PLoS Med. 2022 Jan 4;19(1):e1003858. doi: 10.1371/journal.pmed.1003858 (PMC8726492; doi:10.1371/journal.pmed.1003858)
Supplement: S2 Table — (DOCX) [file pmed.1003858.s004.docx]

### S2 Table: Primary outcome chlamydia testing, intervention group analysis

|  | Removal of incentives only (A) | | | Removal of audit+feedback only (B) | | | Removal of incentives & audit/feedback (C) | | | Control (D) | | |
| --- | --- | --- | --- | --- | --- | --- | --- | --- | --- | --- | --- | --- |
|  | n/N | Testing rate  % (95%CI) | | n/N | Testing rate  % (95%CI) | | n/N | Testing rate  % (95%CI) | | n/N | Testing rate  % (95%CI) | |
| Baseline^a^ | 2124/11196 | 19.0 (16.2 to 21.8) | | 2467/11944 | 20.7 (17.1 to 24.2) | | 2468/11566 | 21.3 (18.8 to 23.9) | | 3050/14819 | 20.6 (17.3 to 23.8) | |
| Year 1 | 1344/10263 | 13.1 (10.7 to 15.5) | | 1641/11717 | 14.0 (11.0 to 17.0) | | 1688/11021 | 15.3 (13.2 to 17.4) | | 2651/15035 | 17.6 (13.2 to 22.0) | |
| Year 2 | 829/ 7196^b^ | 11.5 ( 8.6 to 14.4) | | 918/ 8188^b^ | 11.2 ( 7.7 to 14.7) | | 891/ 7455^b^ | 11.9 ( 9.6 to 14.3) | | 2091/12888^b^ | 16.2 (10.8 to 21.6) | |
| Unadjusted  Year 2 vs baseline  (95% CI) | Diff: -8.2 (-10.4 to -6.0)  OR: 0.5 (0.4 to 0.6) | | | Diff: -9.7 (-12.9 to -6.4)  OR: 0.5 (0.3 to 0.6) | | | Diff: -9.4 (-12.2 to -6.6)  OR: 0.5 (0.4 to 0.6) | | | Diff: -5.4 (-8.2 to -2.5)  OR: 0.7 (0.6 to 0.9) | | |
|  | Treatment effect^c^ | | | Treatment effect^c^ | | | Treatment effect^c^ | | |  | | |
|  | OR (95% CI) | | p value | OR (95% CI) | | p value | OR (95% CI) | | p value |  | |  |
| Baseline^a^ | 0.9 (0.7 to 1.2) | | 0.5508 | 1.0 (0.8 to 1.3) | | 0.7732 | 1.0 (0.8 to 1.2) | | 0.8588 |  | |  |
| Year 1 | 0.7 (0.6 to 0.9) | | 0.0161 | 0.8 (0.6 to 1.1) | | 0.1092 | 0.8 (0.6 to 1.0) | | 0.0574 |  | |  |
| Year 2 | 0.7 (0.5 to 1.1) | | 0.0955 | 0.7 (0.4 to 1.1) | | 0.1328 | 0.6 (0.4 to 1.0) | | 0.0509 |  | |  |
| Treatment effect: (intervention-control) (95%CI) | Diff: -2.8 (-6.4 to 0.8)  OR: 0.7 (0.5 to 1.0) | | 0.0601 | Diff: -4.3 (-8.7 to 0.1)  OR: 0.7 (0.4 to 1.0) | | 0.0317 | Diff: -4.1 (-8.0 to -0.1)  OR: 0.7 (0.5 to 1.0) | | 0.0350 |  | |  |
|  | Adjusted treatment effect ^d^ | | | Adjusted treatment effect ^d^ | | | Adjusted treatment effect ^d^ | | |  | | |
|  | OR (95% CI) | | p value | OR (95% CI) | | p value | OR (95% CI) | | p value |  | |  |
| Baseline^a^ | 1.0 (0.8 to 1.2) | | 0.9869 | 1.1 (0.9 to 1.3) | | 0.4525 | 1.0 (0.8 to 1.1) | | 0.7261 |  | |  |
| Year 1 | 0.7 (0.6 to 0.9) | | 0.0067 | 0.7 (0.6 to 0.9) | | 0.0037 | 0.8 (0.7 to 1.0) | | 0.0721 |  | |  |
| Year 2 | 0.7 (0.5 to 1.1) | | 0.1272 | 0.6 (0.4 to 0.9) | | 0.0125 | 0.7 (0.4 to 1.0) | | 0.0361 |  | |  |
| Treatment effect: (intervention-control) (95%CI) | Diff: -1.8 (-4.9 to 1.3)  OR: 0.7 (0.5 to 1.0) | | 0.0660 | Diff: -3.4 (-7.8 to 1.0)  OR: 0.6 (0.4 to 0.9) | | 0.0247 | Diff: -3.4 (-6.5 to -0.2)  OR: 0.7 (0.5 to 1.0) | | 0.0356 |  | |  |

Diff = Absolute difference. OR = Odds Ratio. n=number tested aged 16 to 29 years; N=number of individuals aged 16 to 29 years attending the clinic. ^a^Baseline = the 12-month period prior to randomisation. Year 1 = 1-12 months after randomisation. Year 2 = 13-24 months after randomisation. ^b^Numerator and denominator less than for Baseline and Year 1 because not all clinics contributed 12 months of data to year 2. ^c^Models account for minimisation variables including annual chlamydia testing rates among 16-29 year olds and number of 16-29 year olds attending the clinic each year. ^d^The fully adjusted model contains patient sex, age group and socioeconomic status of the clinics (continuous) in addition to minimisation variables.
