# Supplementary material for: The impact of removing financial incentives and/or audit and feedback on chlamydia testing in general practice: A cluster randomised controlled trial (ACCEPt-able)
Source: PLoS Med. 2022 Jan 4;19(1):e1003858. doi: 10.1371/journal.pmed.1003858 (PMC8726492; doi:10.1371/journal.pmed.1003858)
Supplement: S3 Table — (DOCX) [file pmed.1003858.s005.docx]

### S3 Table: Distribution of minimisation and socio-economic status variables across clinics by intervention group

1. For all 59 clinics randomised

|  |  | Removal of incentive only – A  n(%)  (N=15 clinics) | Removal of audit/ feedback only – B  n(%)  (N=15 clinics) | Removal of incentives & audit/feedback – C  n(%)  (N=15 clinics) | Control  D  n(%)  (N=14 clinics) | All groups  n(%)  (N=59 clinics) |
| --- | --- | --- | --- | --- | --- | --- |
| Number of 16-29 year olds attending each year | <1000 | 12 (80.0) | 11 (73.3) | 11 (73.3) | 10 (71.2) | 44 (74.6) |
|  | ≥1000 | 3 (20.0) | 4 (26.7) | 4 (26.7) | 4 (28.6) | 15 (25.4) |
| Annual testing rate prior to ACCEPt-able | <0.19 | 8 (53.3) | 8 (53.3) | 8 (53.3) | 8 (57.1) | 32 (54.2) |
|  | ≥0.19 | 7 (46.7) | 7 (46.7) | 7 (46.7) | 6 (42.9) | 27 (45.8) |
| Socio-economic quintile | Q1 | 0 (0.0) | 2 (13.3) | 6 (40.0) | 5 (35.7) | 13 (22.0) |
|  | Q2 | 12 (80.0) | 11 (73.3) | 8 (53.3) | 7 (50.0) | 38 (64.4) |
|  | Q3 | 1 (6.7) | 0 (0.0) | 1 (6.7) | 2 (14.2) | 4 (6.8) |
|  | Q4 | 2 (13.3) | 1 (6.7) | 0 (0.0) | 0 (0.0) | 3 (5.1) |
|  | Q5 | 0 (0.0) | 1 (6.7) | 0 (0.0) | 0 (0.0) | 1 (1.7) |

1. For all 55 clinics included in the analysis^a^

|  |  | Removal of incentive only – A  n(%)  (N=15 clinics) | Removal of audit/ feedback only – B  n(%)  (N=14 clinics) | Removal of incentives & audit/feedback – C  n(%)  (N=13 clinics) | Control  D  n(%)  (N=13 clinics) | All groups  n(%)  (N=55 clinics) |
| --- | --- | --- | --- | --- | --- | --- |
| Number of 16-29 year olds attending each year | <1000 | 12 (80.0) | 10 (71.4) | 9 (69.2) | 9 (69.2) | 40 (72.7) |
|  | ≥1000 | 3 (20.0) | 4 (28.6) | 4 (30.8) | 4 (30.8) | 15 (27.3) |
| Annual testing rate prior to ACCEPt-able | <0.19 | 8 (53.3) | 7 (50.0) | 6 (46.2) | 8 (61.5) | 29 (52.7) |
|  | ≥0.19 | 7 (46.7) | 7 (50.0) | 7 (53.8) | 5 (38.5) | 26 (47.3) |
| Socio-economic quintile | Q1 | 0 (0.0) | 2 (14.3) | 5 (38.5) | 5 (38.5) | 12 (21.8) |
|  | Q2 | 12 (80.0) | 10 (71.4) | 7 (53.8) | 6 (46.1) | 35 (63.6) |
|  | Q3 | 1 (6.7) | 0 (0.0) | 1 (7.7) | 2 (15.4) | 4 (7.3) |
|  | Q4 | 2 (13.3) | 1 (7.1) | 0 (0.0) | 0 (0.0) | 3 (5.5) |
|  | Q5 | 0 (0.0) | 1 (7.1) | 0 (0.0) | 0 (0.0) | 1 (1.8) |

^a^One clinic was missing in Group B, two clinics missing in Group C and one clinic missing in Group D
