# Supplementary material for: The impact of removing financial incentives and/or audit and feedback on chlamydia testing in general practice: A cluster randomised controlled trial (ACCEPt-able)
Source: PLoS Med. 2022 Jan 4;19(1):e1003858. doi: 10.1371/journal.pmed.1003858 (PMC8726492; doi:10.1371/journal.pmed.1003858)
Supplement: Attachment — Submitted filename: Response to reviewers-FINAL.docx [file pmed.1003858.s006.docx]

Re: The impact of removing financial incentives and/or audit and feedback on preventive care activities in general practice: A cluster randomised controlled trial (ACCEPt-able)" (PMEDICINE-D-21-02501R1)

Thank you for considering the above manuscript for publication in PLOS Medicine. We respond to the Editor’s and Reviewers’ comments below. *Please note that the line numbers referred to in our response, are from the copy of the revised manuscript with TRACK changes highlighted*.

Editor:

Noting PLOS' data policy, <https://journals.plos.org/plosmedicine/s/data-availability>, please state whether the study's ethics approval would permit the study data to be shared with other researchers under conditions of confidentiality.

**In the abstract and throughout the paper, please quote p values alongside 95% CI, where available.**

***Response***: We have added in p values for all treatment effects in the Abstract and Main results.

**Please add a new final sentence to the "Methods and findings" subsection of your abstract, which should begin "Study limitations include ..." or similar and should quote 2-3 of the study's main limitations.**

***Response***: We have added the following to the Abstract:

*Study limitations included an unexpected reduction in testing across all groups impacting statistical power, loss of four clinics post randomisation and inclusion of rural clinics only.*

**Please relocate the Author Summary after the Abstract.**

***Response:*** This has been done.

**Throughout the text, please remove spaces from within the square brackets for reference call-outs (e.g., "... on others [4,9,10].").**

***Response: T***his has been done.

**Please remove the information on competing interests and funding from the end of the main text. In the event of publication, this will appear in the article metadata, via entries in the submission form.**

***Response:*** This has been done.

**Please abbreviate journal names consistently in your reference list.**

***Response:*** This has been done.

**Please add a completed checklist for the appropriate CONSORT extension as a supplementary document, labelled "S1_CONSORT_Checklist" or similar and referred to as such in the Methods section. In the checklist, please refer to individual items by section (e.g., "Methods") and paragraph number, not by line or page numbers as these generally change in the event of publication**

***Response:*** This has been done.

*** Reviewer #1:

**1. The authors mention in the discussion that incentives (and perhaps audit/feedback as well) may not have been critical in driving test uptake. If that is the case, why would you then expect a decrease in preventive activities if these were removed (see hypothesis in p. 4, l. 118). It would be helpful if this authors would motivate their hypothesis more based on what is known from previous work in Australia and elsewhere.**

***Response:*** Thank you for raising this. We discuss the evidence about incentives and audit and feedback in our Introduction, but have provided further information about the Australian context and provide further justification for our hypothesis as outlined below:

*…..While some observational data suggest a decline in provider activities and quality of care when incentives are removed [5, 12], other data have shown little impact when removed [13, 14]. There is little information about the removal on provider activities and quality of care in the Australian general practice setting…. (lines 145-149)*

*While there is substantial RCT evidence that audit/feedback improves practice [18], observational data suggest that removing audit/feedback may reverse improvements. However, there is little evidence about the impact of removing audit/feedback on GP activities and quality of care in Australia and to our knowledge, no RCT evidence…. (lines 155-158)*

*……..* *Given the intention of financial incentives and/or audit/feedback is to modify provider behaviour in order to improve quality and/or increase efficiency, our hypothesis was that chlamydia testing would decrease if these strategies were removed, providing the first RCT evidence….(lines 169-172)*

**2. In the discussion the authors mention that the incentives comprised less than 10 percent of GP practices' funding. It would be helpful if this would be mentioned earlier, e.g. in the section 'study design and participants'. In addition, what is the maximum incentive size relative to GP's maximum income (or GP practices revenues)? That would tell the reader something about the actual magnitude of the incentive.**

***Response:*** We provide further information about size of payments in the Methods as suggested by the reviewer. The payments we provided GPs were consistent with other payments GP received for similar activities at the time, but we do not know what the maximum incentive size is relative to GPs maximum income.

*Intervention clinics received: i) financial incentives to individual GPs for each chlamydia test:$5 (AUD) per test for up to 20% of 16-29-year olds tested each year to $8 (AUD) per test for over 40% coverage . These payments were electronically transferred to the clinic each quarter. This amount was consistent with the payment of $6 (AUD) GPs received at the time for completing immunisation schedules and corresponds to an annual payment of about $800 assuming an annual chlamydia testing rate of 20% and an average of 800 patients aged 16 to 29 years attending each clinic per year. This total amount, the payment frequency and electronic transfer methods were consistent with that for other government funded general practice-based incentives at the time [4, 24]….(lines 193-202)*

**3. A general point is that the authors could be a bit more consistent in the use of the terms 'GP' and 'Clinic', e.g. on page 5. It is not always clear if the authors talk about individual GPs or about the clinics that they work in.**

***Response:*** Thanks for highlighting this. We have made changes to the manuscript in order to be more consistent with our language. We have made sure that we use the term GP to refer exclusively to individual GPs. When we use the term clinic, we are referring to the actual clinic.

**4. p. 6, l 157: why the choice for the 67th percentile?**

***Response*:** We selected the 67^th^ percentile for our minimisation algorithm to ensure even distribution across intervention groups by size of clinic because there is some uncertainty about the impact of practice size on quality of patient care. We have added the following to the Methods to justify this decision:

*… (<1000vs≥1000, based on the 67^th^ percentile of patients at each clinic to ensure that groups were evenly distributed within relative smaller and larger clinics because of the potential association of clinic size with patient quality of care [26])….(lines 237-240)*

**5. p. 6, l 170-171: I wonder why the authors used this as the denominator instead of all patients in this age range. Perhaps this has something to do with the fact that GPs in Australia are not paid on a capitation basis (?) and only on a FFS basis, which would imply that they only 'observe' their patients when they present themselves with a health issue?**

***Response:*** The reviewer is correct about the fee-for-service basis of GP remuneration in Australia, as we mention in the Discussion (lines 410-412). We used the number of patients who had at least once consultation (i.e. visited the GP) during the same 12 months as the denominator because the GP can only prescribe the chlamydia test when the patient attends for a health consultation. We haven’t made any changes to the text.

**6. p. 10, l 283: given that the focus was on Chlamydia screening rates, I don't think the authors can say something about 'preventive activities' in general. I think this should be changed in the title of the manuscript as well.**

***Response:*** Thank you, we agree. We have amended the text and have referred specifically to chlamydia testing in the title and throughout the manuscript. We also added the following text to the limitations in our Discussion:

*Fifth, we evaluated the impact of the removal of incentives and audit/feedback on chlamydia testing so our results may not be generalisable to other preventive health activities in general practice…(lines 475-477)*

**7. p. 10, l 305-307: I found this part a bit difficult to follow.**

***Response:*** We apologist for any confusion. We have re-worded this as follows:

*Chance cannot be excluded because we did not expect a reduction in testing in clinics that retained incentives thereby reducing our effective sample size and our observed treatment effect of 0.9% was considerably smaller than our hypothesised 5%. (lines 412-415)*

***** Reviewer #2:**

**1) In the statistical analysis section on page 7, it says "our analysis was a modified intention-to-treat as clinics that were unable to provide outcome data at trial end were excluded from the primary analysis". However, it's either ITT or not ITT, the wording 'modified ITT' is vague and widely criticised so should be avoided. This is essentially a complete case analysis as 4 clusters/clinics were dropped and excluded in the primary analysis.**

**Response:** Thanks for drawing this to our attention. We agree and have amended the text as follows:

*We aimed to compare the groups according to intention-to-treat, but in a change to the published protocol [21], we had to exclude clinics that were unable to provide outcome data at the end of the trial from the primary analysis. (lines 281-283)*

**2) Analyses. As 4 clusters/clinics were dropped and excluded in the analyses, the randomisation were broken and interupted at both cluster and patients levels, therefore the primary analyses only adjusted for two minimisation factors are not sufficient and inadequate as clearly we can see the imbalance at the cluster level in socioeconomic status. Instead, fully-adjusted analyses should be used for primary and all analyses throughout the paper to avoid potential bias due to the loss of 4 clusters.**

***Response:*** Thank you for raising this point. The imbalance in socioeconomic status only applied to the intervention group analysis – not our primary factorial analysis. However, we agree with the reviewer that the loss happened post-randomisation. We have amended the text and now list the results of the fully adjusted models only in the Results section. Both the primary analysis, adjusted for minimisation variables only, and the fully adjusted analysis (adjusted for the minimisation variables, patient age, gender and socio-economic status of clinics) are provided in Table 2. The Results text has been changed as follows:

*Baseline characteristics at the patient and cluster-level were similar between pairs of intervention groups (for factorial analysis) (Table 1), but given the loss of four clinics, we report only the results from the fully adjusted models in the text. Results from the model adjusted for minimisation variables only and the fully adjusted model (adjusted for minimisation variables and patient age and gender and socio-economic status of the clinic) were similar (Table 2). (lines 329-333)*

**3) Missing data. Normally, a complete case analysis will go alongside with a sensitivity analysis with missing data imputation. However, the missing data issue was not dealt with at all in this study. While, it may not be feasible in this study for missing data imputation as 4 clinics were dropped, but it would be very useful to compare the characteristics of dropped clusters with that of remaining clusters in the same arm to see whether it's missing at random or not at random so that make sure we are able to address the potential bias and impact of the missing data on the trial results.**

***Response:*** Thank you for raising this point. We did not do additional analyses to explore the sensitivity of the results to exclusion of clinics without accessible data. These clinics did not have data because of technical issues with our data extraction tool and their medical records software –their exclusion was nothing to do with the interventions nor chlamydia testing. The characteristics (minimisation variables and socio-economic status) of the excluded clinics were similar to the those for the clinics with accessible data. We have added in two additional supplementary tables showing that exclusion of the four clinics had little impact on the distribution of minimisation and socio-economic status variables across all groups (Supplementary Tables 3A and 3B).

We have also provided further information about potential limitations in our Discussion as follows:

*Third, four clinics did not provide testing data and were excluded from the analysis after randomisation. However, their removal had little impact on the distribution of minimisation and socio-economic variables across the intervention groups and these variables were adjusted for in our analysis minimising any bias. (line 469-472)*

*** Reviewer #3:

**General Comments**

**This appears to be a revision. I was not one of the initial reviewers.**

***Response:*** We would like to clarify that the version R1 included only changes that the Editors asked for before sending for peer review. The Editors assessed our initial submission to decide whether or not to submit for external peer review. We were then invited to submit the manuscript formally with the additional necessary meta-data.

**I have several major concerns with the manuscript and analysis. First, the analytic method, the authors' own definition of clinical significance, and the marginal nature of the results call the conclusions into question. The protocol said that "analyses will be adjusted for the chlamydia testing rate at each general practice immediately prior to commencing ACCEPt-able" and "account for cluster…GP and patient variability." In the actual analysis, the investigators adjusted for clinic clustering, annual chlamydia testing rates, and the number of 16 to 29-year-olds attending the clinic each year. A more fully-adjusted model included patient sex, age-group, and socio-economic status of the clinic. Only the less-adjusted model examining the impact of the removal of audit and feedback was of marginal statistical significance, and even here, the odds ratio - which the authors identify as the planned primary analysis (page 7, line 202) - includes 1.0. Further adjustment yielded a non-significant result.**

***Response:*** We apologise for any confusion. We did include a fully adjusted model that included each of these variables in the model. Both our primary analysis model and fully adjusted model had very similar results. As stated in our Methods and indicated in the footnotes of the results tables:

*A logistic model generated odds ratios and absolute differences were obtained from a model with an identity link function with binomial error distribution. These models also provided 95% confidence intervals and p-values and adjusted for minimisation variables only (annual chlamydia testing rates among 16-29 year olds in the clinic and number of 16-29 year olds attending the clinic each year) as is recommended.[29] We also obtained the results of an adjusted model post-hoc that in addition to the minimisation factors, also included the variables that were adjusted for in the ACCEPt trial (patient sex and age-group and socio-economic status quintile of the clinic – ‘fully adjusted model’).[19, 30] (lines 288-295)*

**In the Methods, the investigators cited a 5% absolute reduction as a "clinically relevant result." (I have actually used this exact clinically significant difference in some of our own analyses of quality or health services research.) The absolute reduction was only a 3.1% absolute decline. In all, this does not seem strong enough to hang the conclusion that "financial incentives don't work, but audit and feedback does so primary care practices should invest in audit and feedback." (See "Third" below regarding overgeneralizing the result.)**

***Response:*** Thanks for raising this. A reduction of the level reported in our paper would translate into about 160,000 people not being tested which is of considerable public health importance in the Australian population setting. However, we take on board the reviewer’s comments and have modified our Conclusion as follows (the key changes are in bold text below):

*In this cluster RCT,* ***we found that the incentive offered had little impact on chlamydia testing in Australian general practice****. The total amount of financial incentive payments received per year in our trial was consistent with other incentive payments GPs received at the same time in Australia.* ***It is possible that the removal of financial incentives might have a greater impact if incentive payments were to make up a greater proportion of GP income****, such as in the UK. RCT evidence is needed to investigate this question. The removal of audit and feedback with a face-to-face visit resulted in a relative reduction in activity of 30% overall. A reduction of this level could have considerable public health impact at the population level* ***with fewer chlamydia tests conducted and infections undetected****. Our results suggest that in Australia at least,* ***audit and feedback is an important intervention for influencing GP behaviour for preventive health activities like chlamydia testing****. The use of digital platforms that include automated reports and online communication could reduce the costs associated with audit and feedback. Our finding that chlamydia testing also decreased in clinics that retained incentives and audit and feedback, highlights that simply retaining these interventions over time is not enough, further studies should investigate how to sustain clinician behaviour change over time. (lines 482-500)*

**Second, crucial details of GPs workflow around chlamydia testing are not described that might help the reader understand the lack of effectiveness of either the ACCEPt intervention or the persistence of financial incentives or audit and feedback. The reader is left to wonder what it is about ordering Chalmydia testing that is so difficult that an intensive, multifaceted intervention only led to a 7% increase in testing rates and those rates reverted almost back to their pre-ACCEPt level. For behavioral interventions, details of workflow and interventions are extremely important (Fox et al. BMJ. 2020;370:m3256), perhaps especially when they are not successful. It is overly simplistic to say "financial incentives and audit and feedback don't work." Regarding chlamydia testing, how, by whom, and when was it done? Regarding the financial incentives, in what context were they delivered (i.e., was it an unrecognizably small part of some larger, quarterly payment or was it identifiable as a "chlamydia testing incentive"). Regarding the feedback, all we know is that it was "given to GPs during a [quarterly] visit with a research officer," but how was it delivered? Was it provided with a descriptive norm or injunctive norm? Lack of these details makes it hard for readers to learn anything from the ACCEPt and ACCEPt-able experience.**

***Response:*** Thanks for raising these points. We are unsure about the 7% cited by this reviewer and wonder if it was a typo. In ACCEPt, testing increased by 11.9% (from 8.2% to 20.1%) which was a relative increase of 70% in the intervention group compared to the control group. It is very interesting however, chlamydia testing rates declined during *ACCEPt-able* across all intervention groups suggesting it is challenging to sustain provider behaviour change over time. We did comment about this in our Discussion. Chlamydia testing in general practice can be difficult to do because it is a sensitive topic to raise during a consultation, particularly if the consultation is for a non-sexual health related issue. There is abundant literature discussing the challenges associated with chlamydia testing in primary care. We have not discussed this further in this paper because our paper aimed to investigate the impact of removing interventions, not exploring what is needed to increase chlamydia testing. However, as requested, we have provided additional information about our intervention and how chlamydia testing worked in the clinics in our Methods as follows under the Study design and participants and Interventions headings (the key changes are in bold text below):

Study design and participants

***At the time of ACCEPt, opportunistic chlamydia testing was recommended annually for sexually active 16 to 29 year olds in general practice [20]. How chlamydia testing was conducted varied between clinics with some clinics using GPs to initiate testing and others using practice nurses; some using clinician-collected specimens for testing, others allowing patients to self-collect specimens (e.g. urine specimens or high vaginal swabs) and leave them at the clinic for testing while others required the patient to attend an external pathology collection centre for testing.*** *Intervention clinics received: i) financial incentives to individual GPs for each chlamydia test:$5 (AUD) per test for up to 20% of 16-29-year olds tested each year to $8 (AUD) per test for over 40% coverage . These payments were electronically transferred to the clinic each quarter. This amount was consistent with the payment of $6 (AUD) GPs received at the time for completing immunisation schedules and c****orresponds to an annual payment of about $800 assuming an annual chlamydia testing rate of 20% and an average of 800 patients aged 16 to 29 years attending each clinic per year.*** *This total amount, the payment frequency and electronic transfer methods were consistent with that for other government funded general practice-based incentives at the time [4, 24]. ii) Audit/feedback where individual GPs were provided with a one-page report* ***that summarised each GP’s chlamydia testing rates for the previous quarter including the number of patients aged 16 to 29 years who had consulted them, the number they tested and the number that tested positive. The report also included a statement of the total amount of incentive payments they would receive for that quarter’s testing.*** *The report was given to individual GPs during a quarterly face-to-face visit with a research officer who explained the results and worked with the GP to identify strategies to help increase their testing rates. The intervention also included chlamydia education* ***(hard-copy and online resources about chlamydia and its management that was given to all GPs and nurses in a face-to-face meeting with a research officer after randomisation) and computer alerts prompting testing. Not all clinics used the computer alerts. Guided by normalisation process theory, a member of the research team worked with each clinic to tailor the intervention to the resources of the clinic and identify strategies to facilitate testing and embed it into routine practice [25].*** *(lines 187-215)*

Interventions

*All GPs within each clinic received the same intervention. The groups receiving audit/feedback received the same one-page report as for the ACCEPt trial that summarised each GP’s chlamydia testing rates for the previous quarter and included a statement of the total amount of incentive payments they would receive for that quarter’s testing. The report was given during a quarterly face-to-face visit with a research officer who explained the results and worked with the GP to identify strategies to help increase their testing rates. (lines 248-254)*

Thank you for suggesting the reference by Fox and colleagues. We have also added the following to the Discussion:

*We provided clinics with the same level of support during ACCEPt-able, but we did not monitor whether there were changes in their use of other strategies to facilitate testing such as using computer alerts, and while new GPs received our chlamydia educational package, we did not provide any further educational support to already participating GPs. The lack of ongoing ‘calibration’ of the intervention and its support may have contributed to declining testing rates across all groups [38]. (lines 427-433)*

I **am also curious about the details of how financial incentives and audit and feedback were deimplemented. Practices were invited and had to consent to participate in this deimplementation RCT. As such, presumably they were told they were going to have a prior intervention removed ("A research officer informed clinics of their allocation"), but how was this done? Thus, the invitation, consent, and enrollment process must have included the implicit message that "Chlamydia testing is less important than it was before" or "we are not going to be monitoring Chlamydia testing as closely." Just as the Hawthorne effect in an intervention trial is often the most impactful part of the interventions, as part of deimplementation, these implicit messages could have had as big an effect as the actual removal of the interventions. Indeed, most of the decrease in Chlamydia testing occurred between the Baseline and Year 1. Also, what happened to the computerized alerts? Did they persist?**

***Response***: Clinics were informed about *ACCEPt-able* in a face to face meeting with a research officer and provided informed consent before they were randomised. While it is possible that some may have considered that chlamydia testing is less important than before, this is unlikely because there was no change in chlamydia testing guidelines during this time. Clinics were aware we would continue to monitor their chlamydia testing data during *ACCEPt-able*. Not all clinics elected to nor were able to use the computer alert in ACCEPt. We have provided the following additional information in the Methods:

*At the conclusion of ACCEPt, a research officer met with GPs in each intervention clinic, informed them about ACCEPt-able, invited them to participate and obtained informed consent [21]. (lines 218-220)*

*Randomisation took place after clinics were recruited into ACCEPt-able and consented to participate. (lines 241-242)*

**With apologies for 1 paragraph of editorializing, given the findings of ACCEPt and ACCEPt-able, I would guess the clinic environment and the mechanism of ordering was reliant on busy GPs to remember to bring up, discuss, and order Chlamydia testing that was not integrated into regular work-flow. Financial incentives and audit and feedback have their place in nudging clinicians regarding complex decision-requiring behaviors. But for something as simple as Chlamydia testing, a much better solution is to use practice facilitation to systematize or routinize the activity and remove it from the need for the GP to remember and act. In my own health system, we achieve rates around 90% when we have our check-in or rooming staff systematically perform tasks and use standing orders like for influenza vaccination, fall screening, depression screening, and tobacco screening.**

***Response:*** Thanks for mentioning these strategies and experiences. General practice in Australia is a dynamic environment and the strategies for managing chlamydia testing vary considerably between and even within different clinics. Each clinic uses its receptionists and nursing staff differently and not all clinics have nursing staff. As mentioned in response to a comment by reviewer 3 above, it is difficult to increase chlamydia testing in general practice and that is why we used a multifactorial intervention to increase testing as part of the ACCEPt trial. Chlamydia testing rates increased by an additional 70% in intervention clinics in ACCEPt compared with the control group which received no interventions at all. *ACCEPt-able* provided the unique opportunity to explore the impact of removing audit/feedback and the incentive payments providing the first RCT evidence. We haven’t made any further changes to the text.

**Third, related to the marginal nature of the results and details around practice and interventions, it is an overgeneralization to say that these interventions did or did not affect "preventive care in general practice." Because the details of workflow are crucial, all that has been shown is that "the removal of financial incentives or audit and feedback did not affect Chlamydia testing in rural Australian general practices." While that might be overly narrow, the details around individual preventive services, like Chlamydia testing, cannot be generalized to ALL preventive services (e.g., counseling, cancer screening, immunizations, etc.).**

***Response:*** Thanks for this comments. We have amended our conclusion as outlined above in response to an earlier comment by Reviewer 3 and changed the focus from preventive services to chlamydia testing throughout.

**Fourth, given that the interventions were delivered on a quarterly basis, it is unclear why the investigators chose to analyze their data on an annual basis and, in the analysis, only compare Year 2 to the baseline year. This effectively ignores all the data from Year 1 (when more practices were participating) and treats all of the data collected during Year 2 as equivalent (i.e., a visit in Month 13 is the same as a visit in Month 14). It also ignores the possibility that changes in Chlamydia screening among the groups could have had different trajectories and could have overweighted the contribution of clinics that participated in the trial longer (the analysis was adjusted for clustering by practice, but not by practice volume).**

***Response:*** Thank you for raising this question. We chose to analyse the data on an annual basis because at the time of the trial, annual chlamydia testing was recommended. All data collected in the 12 months prior to randomisation and 24 months post-randomisation were included in the statistical analysis model. This model did not assume or impose a trajectory over time. The treatment effect of primary interest was the change at 13-24 months post-randomisation from the 12-month period prior to randomisation to capture the full effect of removal of incentives or removal of audit/feedback. The intervention period ranged from 0.2 years to 2 years with a mean duration of 1.5 years (SD 0.4). A total of 52 (95%) clinics contributed data to Year 2 (13-24 months post-randomisation). The average duration of the intervention period was similar between groups (1.5 years for Groups A and C; 1.6 years for Groups B and D), therefore all groups were assumed to be affected similarly by duration of the intervention. We haven’t made any further changes to the text.

**Specific Comments**

**Title: As noted above, including "preventive care activities" in the title regarding an intervention that had to do with Chlamydia screening is an overgeneralization.**

***Response:*** We have amended the title as recommended by Reviewer 1 above.

**Page 3, Line 66: Here, and throughout the manuscript, the investigators never state the number of GPs or the number of GPs clustered within practices. I see there were 305 GPs in the 63 clinics randomized to the interventions in the original ACCEPt Trial.**

***Response:*** We have added information about the number of GPs in each intervention group to Table 1. Please note that the 305 GPs reported in the ACCEPt paper refers to the number of GPs who were recruited at baseline. Australian general practice is very dynamic with new GPs joining clinics throughout *ACCEPt-able.* The data reported in Table 1 refers to the number of GPs who were recruited throughout *ACCEPt-able*.

**Page 3, Line 71: More a problem with presentation, here and elsewhere, it is confusing that the authors present the RCT as organized in 4 groups, but then only analyze the data in 2 groups. This can be improved by mentioning the analytic plan earlier on and, in Lines 77 through 84, introducing the actual comparison groups much earlier in the sentences. As written, one only discovers the group being discussed in the middle of the sentences, after some data about those groups has been presented.**

***Response:*** We apologise for any confusion. We have added the following text to the Abstract to provide further clarification:

*We undertook a factorial analysis in which we investigated the effects of removal versus retention of incentives (Groups A+C versus Groups B+D) and the effects of removal versus retention of audit/feedback (Group B+C versus Groups A+D) separately. (lines 78-81)*

And have added the following text to the Statistical analysis section:

*We conducted a ‘factorial analysis’ as our primary analysis. This investigated the effects of removal versus retention of incentives (Groups A+C versus Groups B+D) and audit/feedback (Groups B+C versus Groups A+D) separately on annual chlamydia testing over two years. (lines 277-280)*

In the Results section and Table 2, we have clearly indicated which Groups are involved in each analysis.

**Page 3, Abstract General Comment: The timeframe of the assessment of the primary outcome is not stated (in Year 2 after randomization).**

**Response:** We have added the following to the Abstract and also specified the time frame in the Statistical analysis section as follows:

**Abstract:**

*Clinics were recruited between July 2014 and September 2015 and were followed for up to two years or till end of December 2016. (lines 69-70)*

**Methods:**

*Clinics were followed for up to two years or till the end of December 2016, whichever came first. (lines 230-231)*

**Statistical analysis:**

*We conducted a ‘factorial analysis’ as our primary analysis. This investigated the effects of removal versus retention of incentives (Groups A+C versus Groups B+D) and audit/feedback (Groups B+C versus Groups A+D) separately on annual chlamydia testing over two years…(lines 277-280)*

*…..For each intervention, we fitted generalised linear models, using generalised estimating equations to account for clustering at clinic-level and assessed the impact of the intervention on chlamydia testing in year two compared with baseline. (lines 285-288)*

**Page 5, Line 123: The authors say there were no changes to the trial methods, but later report a protocol deviation (modified intention-to-treat analysis).**

***Response:*** Thank for your raising this point. We have provided some further clarification around this as outlined below. We have modified our comment about the modified intention to treat analysis based on our response to reviewer 2 above:

**Methods:**

*There were no changes to trial recruitment, implementation, management or follow up methods, but in a change to the published protocol, we had to exclude clinics that were unable to provide outcome data at the end of the trial from the primary analysis (further detail provided below). (lines 178-181)*

**Statistical analysis:**

*We aimed to compare the groups according to intention-to-treat, but in a change to the published protocol [21], we had to exclude clinics that were unable to provide outcome data at the end of the trial from the primary analysis. (lines 281-283)*

**Page 5, Line 136: The authors mention, in addition to financial incentives and feedback, computer alerts, but what about the educational package? That was part of the initial multicomponent ACCEPt intervention. Was that only done at the beginning of ACCEPt and never repeated?**

***Response:*** The educational package included hard copy and online resources providing information about chlamydia and its management and information that could be given to patients. It was provided to all GPs in a face to face meeting with a research officer when they were recruited into ACCEPt. New GPs joining the clinics during *ACCEPt-able* were recruited and provided with the educational package. We have provided further comment about the educational package in the Methods and Discussion as follows:

**Methods:**

*The intervention also included chlamydia education (hard-copy and online resources about chlamydia and its management that was given to all GPs and nurses in a face-to-face meeting with a research officer after randomisation) and computer alerts prompting testing. (lines 209-212)*

*During ACCEPt-able, we recruited and consented new GPs who were also provided with the chlamydia education package. (lines 228-230)*

**Discussion:**

*We provided clinics with the same level of support during ACCEPt-able, but we did not monitor whether there were changes in their use of other strategies to facilitate testing such as using computer alerts, and while new GPs received our chlamydia educational package, we did not provide any further educational support to already participating GPs. (lines 427-431)*

**Page 5, Line 143: If a patient reported risk factors or genital symptoms and required further testing, how were repeat patients counted? How were they handled in the analysis?**

**Response:** Thank you for raising this question. As stated in the definition of the primary outcome, only one test per person in any 12 month period was counted in our primary outcome - t*he primary outcome was annual chlamydia testing rate among 16-29-year olds attending the clinic. The numerator was the number of people aged 16-29 years who had at least one test within 12-months; the denominator was the number aged 16-29 years who had at least one consultation during the same 12-months (lines 257-260).* Our data extraction tool extracted consultation and testing data including a unique non-identifying patient code – this allowed us to account/adjust for multiple tests and consultations per patient in our analysis. No changes have been made to the text.

**Page 6, Line 168: The authors state that the primary outcome was the "annual chlamydia testing rate," but do not say when this was assessed. It is not until the reader gets to Table 2 does it become clear that the primary analysis is between Baseline and Year 2.**

***Response:*** We have provided additional information in our Methods to make this clear in our analysis.

*For each intervention, we fitted generalised linear models, using generalised estimating equations to account for clustering at clinic-level and assess the impact of the intervention on chlamydia testing in year two compared with baseline. (lines 285-288)*

**Page 8, Line 243: The authors need to define the interpretation and direction of the interaction effect. Was the 3.2% (NS) the marginal increase in screening in practices randomized to retain both of the interventions relative to practices retaining neither? How is this conceptually different from the analysis presented on page 9, line 260?**

**Response:** The interaction effect means that the effect of removing audit/feedback on chlamydia testing does not vary significantly between whether clinics received or did not receive incentive payments and vice versa. The direction is that clinics which retained incentive payments had a 3.2% larger decrease in testing from baseline to year 2 by removal of audit/feedback than those where incentives were removed.

Page 9, line 260 describes the results of the “intervention group” analysis presented in Supplementary Table 2 which compares each intervention group (Groups A, B or C) with the control (Group D which retained incentives and audit/feedback) separately; it doesn’t assess for example, whether the treatment effect of removing incentives varies by whether or not audit/feedback is removed or retained. We haven’t made any further changes to the manuscript.

**Page 9, Line 274: Given that all of the clinics participating in ACCEPt and ACCEPt-able were rural, it is not clear how information about metropolitan clinics is included.**

**Response: T**hanks for highlighting this omission. We have added the following to the Cost and consequences section of the Methods:

*As the trial was based in rural clinics, we conducted a sensitivity analysis to examine the potential costs and consequences for removal or retaining the interventions in metropolitan clinics where travel costs and staff time for travel are likely to be reduced considerably. (lines 313-316)*

**Page 18, Table 1: The table should include information about GPs within practices (central tendency and variation).**

***Response:*** We have provided further information about the number of GPs within intervention groups in Table 1.

**Page 20, Table 2: In the footnote and elsewhere, the authors say the denominator is "N=number of individuals attending the clinic," but I think it should be the number of 16-29-year-olds.**

***Response:*** Yes, it does refer to the number of 16 to 29 years olds. This has been added to the footnotes.

**Page 21, Table 3: The rows about "authorising payments" are unclear. What do these represent?**

***Response:*** We have modified the text in Table 3 as follows:

*Program-related activities and resources: Labour and technical support to collate data for dispensing incentive payments to clinics*

***

We have made some additional minor editorial changes to the text highlighted in the track changes. I have uploaded a clean and marked copy of the manuscript and look forward to hearing from you in due course.

Thanks and kind regards

Jane Hocking
