# Supplementary material for: The impact of removing financial incentives and/or audit and feedback on chlamydia testing in general practice: A cluster randomised controlled trial (ACCEPt-able)
Source: PLoS Med. 2022 Jan 4;19(1):e1003858. doi: 10.1371/journal.pmed.1003858 (PMC8726492; doi:10.1371/journal.pmed.1003858)
Supplement: Attachment — Submitted filename: Response to reviewers - R2.docx [file pmed.1003858.s007.docx]

Re: The impact of removing financial incentives and/or audit and feedback on preventive care activities in general practice: A cluster randomised controlled trial (ACCEPt-able)" (PMEDICINE-D-21-02501R2)

Thank you for considering the above manuscript for publication in PLOS Medicine. We respond to the Editor’s and Reviewers’ comments below. *Please note that the line numbers referred to in our response, are from the copy of the revised manuscript with TRACK changes highlighted*.

**Requests from Editors:**

**1)	The Data Availability Statement (DAS) requires revision. If part of the data is not freely available, please include an appropriate contact (web or email address) for inquiries (this cannot be a study author/ co-author).**

***Response:*** We have modified the text provided as follows:

*Data cannot be shared publicly because ethics approval was not provided to make the data available. Ethics committee approval will be required for access to the original data from this trial. Inquiries about data access can be made to RACGP National Research and Evaluation Ethics Committee (ethics@racgp.org.au).*

**2)	Please place the Author Summary after the Abstract.**

***Response:*** The Author Summary is after the Abstract.

**3)	Abstract - In the last sentence of the Abstract Methods and Findings section, please describe the main limitation(s) of the study's methodology.**

***Response:*** We apologise for any confusion, but we had already included the main limitations in the abstract. Have outlined them below (see lines 55-57):

*Study limitations included an unexpected reduction in testing across all groups impacting statistical power, loss of four clinics post randomisation and inclusion of rural clinics only.*

**4)	The terms gender and sex are not interchangeable (as discussed in** <http://www.who.int/gender/whatisgender/en/> ); please use the appropriate term.

***Response:*** Thank you for raising this. We have replaced the word gender with sex in two places in the manuscript.

**5)	Please indicate in the figure captions the meaning of the bars and whiskers in the figures**

***Response:*** This has been added as a footnote to the captions for the figures.

**6)	Please remove the information on funding acquisition from the end of the main text. In the event of publication, this will appear in the article metadata, via entries in the submission form**

***Response:*** This has been removed

7)	References:

a)	Please ensure there is no space between in-text reference call outs. For example, “…community [2,8,9].”

b)	Please ensure that journal name abbreviations consistently match those found in the National Center for Biotechnology Information (NCBI) databases. <https://journals.plos.org/plosmedicine/s/submission-guidelines#loc-references>.

***Response:*** This has been done.

**8)	To help us extend the reach of your research, please provide any Twitter handle(s) that would be appropriate to tag, including your own, your coauthors’, your institution, funder, or lab.**

***Response:*** Our Twitter handles include:

@JHocking01

@unimelbMSPGH

@SexHealthUoM

@nicolamlow

**Reviewer #2**

**Many thanks authors for their great effort to improve the manuscript. I am mostly satisfied with the response and revision. However, one minor issue still remains. In response to my comments on missing data, the authors said "We have added in two additional supplementary tables...(Supplementary Tables 3A and 3B)". However, these two tables have neither been mentioned in the final clean version nor appeared anywhere in the supplementary information. Could authors please add and link these two supplementary tables in the submission? Also, it seems a previous version rather than a clean version was presented in the resubmission. Can authors make sure all the changes are included and appear in the final clean version?**

***Response:*** I am very sorry about this – I must not have uploaded the documents correctly and I note that I hadn’t referenced the tables in the text. They are referenced at line 420-421 in the manuscript.

**Reviewer #3:**

**This is a revised version. The "clean" version that was uploaded appears to be the same as the original (R1) revision. I am reviewing the response letter and the "marked" version. Not having the clean version has made this review more challenging.**

***Response:*** Once again, I am very sorry about this.

**The authors were non-responsive to my first comment about using multiple models and the lack of statistical significance of the results as shown in Table 2 (the OR for all treatment effects include 1.0).**

***Response:*** We apologise for not explicitly addressing this in our earlier response. We presented both the absolute difference in testing between groups (for which the 95%CI excluded “0”) and the odds ratios in Table 2. The odds ratios did include 1.0 due to rounding up.

**With apologies, for my fourth point about the analysis only comparing year 2 in aggregate to the baseline year, included a typo. I meant to write that "a visit in Month 13 is the same as a visit in Month 24" (not "Month 14"). The authors were non-responsive on this point. At a minimum, they need to address this as a limitation.**

***Response:*** We apologise for any confusion in our earlier response. As we mentioned, all data collected in the 12 months prior to randomisation and 24 months post-randomisation were included in the statistical analysis model. This model did not assume or impose a trajectory over time. The treatment effect of primary interest was the change at 13-24 months post-randomisation from the 12-month period prior to randomisation to capture the full effect of removal of incentives or removal of audit/feedback. The intervention period ranged from 0.2 years to 2 years with a mean duration of 1.5 years (SD 0.4). A total of 52 (95%) clinics contributed data to Year 2 (13-24 months post-randomisation). It was reassuring that the average duration of the intervention period was similar between groups, therefore all groups were assumed to be affected similarly by duration of the intervention. However, we have added the following as a limitation to the Discussion (lines 423-426).

*Fifth, we assessed the impact of the intervention on chlamydia testing in year two compared with baseline and not all clinics remained in the trial till end of year 2. However, it was reassuring that the duration of the intervention period was similar between groups.*

Once again, thank you very much for considering our manuscript.

Kind regards

Jane Hocking
